# Modified triple pelvic osteotomy for adult symptomatic acetabular dysplasia: clinical and radiographic results at midterm follow-up

**DOI:** 10.1186/s13018-018-0922-y

**Published:** 2018-09-15

**Authors:** Jiajun Wu, Yang Yang, Xiuhui Wang, Xiaoxiao Zhou, Changqing Zhang

**Affiliations:** 1 Department of Orthopedics, Zhoupu Hospital Affiliated to Shanghai University of Medicine & Health Sciences, No. 1500 Zhouyuan Road, Pudong New Area, Shanghai, 201318 China; 2 Department of Orthopedics, Taizhou Hospital Affiliated to Wenzhou Medical University, Zhejiang, China; 3 Department of Orthopedics, Shanghai Sixth People’s Hospital Affiliated to Shanghai Jiao Tong University, No. 600 Yishan Road, Xuhui District, Shanghai, 201306 China

**Keywords:** Modified triple pelvic osteotomy, Symptomatic acetabular dysplasia, Tönnis grade, Harris score, Radiographic outcomes

## Abstract

**Background:**

Acetabular dysplasia is the most common cause of secondary arthritis of the hip joint. Achieving maximum restoration of the acetabular coverage and medialization of the femoral head remains difficult with the original Steel triple pelvic osteotomy for acetabular dysplasia in children and adults. This study intended to answer the following questions: (1) Are the midterm functional results of our modified procedure favorable, particularly in relation to Harris scores? and (2) On the basis of the Tönnis grade, does this procedure has a different effect on radiographic parameters and functional results at midterm follow-up?

**Methods:**

This study included 26 consecutive adult patients with symptomatic acetabular dysplasia (28 hips) who underwent modified triple pelvic osteotomy through two incisions between July 2005 and June 2012. According to the preoperative Tönnis grade, the patients were divided into T0 (Tönnis grade 0), T1 (Tönnis grade 1), and T2 (Tönnis grade 2) groups. Wiberg center-edge (CE) angle, Sharp acetabular angle, lateralization, and Harris scores were analyzed to assess the radiographic and clinical outcomes.

**Results:**

The mean CE angle (28.43° [± 3.58°], *p* < 0.05), Sharp acetabular angle (36.39° [± 3.26°], *p* < 0.05), lateralization (16.82 mm [± 3.10 mm], *p* < 0.05), and Harris scores (89.07 [± 4.97], *p* < 0.05) at the last follow-up significantly improved compared to those preoperatively. Multiple comparisons of radiographic outcomes among the three groups indicated no significant difference (*p* < 0.05). Harris scores in group T2 were significantly lower than those in groups T0 (*p* < 0.05) and T1 (*p* < 0.05). No major complication was observed.

**Conclusions:**

Our modified triple pelvic osteotomy for adult symptomatic acetabular dysplasia with early-stage osteoarthritis could lead to excellent radiographic outcomes, good clinical results, and lower complication rates.

## Background

Acetabular dysplasia with a poorly developed acetabulum and insufficient femoral head coverage is the most common cause of secondary arthritis of the hip joint [1]. The Steel triple pelvic osteotomy for acetabular dysplasia has become widely used in either children or adults to restore hip joint biomechanical properties, relieve hip joint symptoms, and alter osteoarthritis development since Steel described it in 1973 [2]. The major drawback with the original Steel triple pelvic osteotomy is the difficulty in achieving maximum restoration of the acetabular coverage and medialization of the femoral head, resulting in an unpredictable prognosis [3–7]. Various modified triple pelvic osteotomies as a treatment for adult acetabular dysplasia have been developed with the intent of achieving favorable clinical results [8–12].

This study aimed to review our series of patients treated with modified triple pelvic osteotomy for adult acetabular dysplasia and describe a variation in this procedure. The following questions were raised: (1) What are the midterm functional results of this procedure, particularly in relation to Harris scores? and (2) On the basis of the Tönnis grade, does this procedure have a different effect on radiographic parameters and functional results at midterm follow-up?

## Methods

### Patients

This study included 28 consecutive patients aged > 18 years (30 hips) who had symptomatic acetabular dysplasia on standard pelvic radiography accompanied by early-stage osteoarthritis and underwent modified triple pelvic osteotomy at our institution between July 2005 and June 2012. Patients with acetabular dysplasia secondary to Down syndrome, inflammatory arthritis, Legg–Calvé–Perthes disease, neuromuscular conditions, and associated severe arthritis were excluded. One patient who was lost to follow-up and another who lacked complete radiographic data were excluded. Finally, 26 patients (28 hips) were included in this study. This study was approved by the institutional review board of Shanghai Sixth People’s Hospital, and informed consent was obtained from all patients.

### Methods

#### Surgical technique

##### Step 1: Exposure and osteotomy

**Fig. 1:**
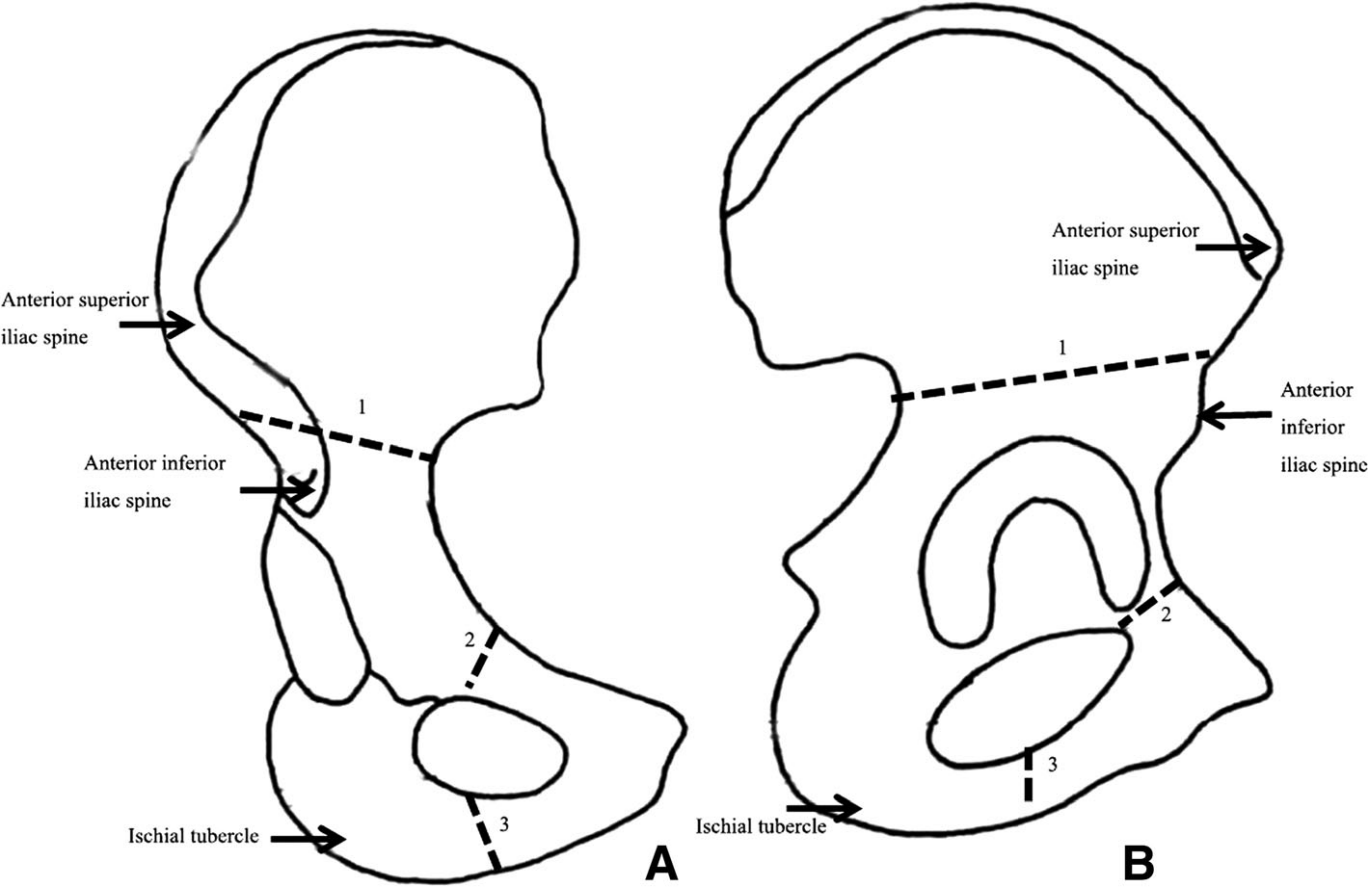
Diagram showing the **a** anteroposterior and **b** lateral views of the surgical technique. 1, iliac osteotomy; 2, pubic osteotomy; and 3, ischial osteotomy

The procedure was performed through two incisions, with all patients under general anesthesia and in the supine position. The first incision (approximately 10 cm) was created along the anterior superior iliac crest to expose the iliac crest; the iliacus and gluteal muscles were bluntly detached to expose the greater sciatic notch. A jigsaw was placed through the greater sciatic notch using a customized tangential clamp, and iliac wing osteotomy was then performed. The second incision (approximately 5 cm) was created on the medial surface of the proximal thigh, located in the middle third of the medial thighs and parallel to the inguen through the space between the adductor brevis and adductor longus muscles; osteotomy of the ischial and pubic rami was completed (Fig. 1).

##### Step 2: Restoration and fixation

After completing the three osteotomies, the acetabulum was mobilized to provide improved anterior and lateral coverage of the femoral head, which required rotation and traction of the limb under radiographic monitoring. Two 3.5-mm K-wires were inserted into the iliac osteotomy gap above the iliac wing, and a pre-bent locking reconstruction plate (DePuy Orthopaedics, Inc., Warsaw, IN, USA) was also used to fix the osteotomy fragments. Moreover, allograft (cortical–cancellous blocks; Osteorad Biological Materials Co., Taiyuan, China) was placed on the iliac osteotomy sites. Finally, the correction of CE angle and Sharp acetabular angle was estimated using the anteroposterior pelvic radiograph after the fixation with C-arm fluoroscopy.

##### Step 3: Closing and rehabilitation

A negative pressure drainage tube was placed into the first incision prior to its closure. After the surgery, no immobilization and traction were required. Three days later, patients were instructed to perform hip and knee flexion exercises. At 3 months after surgery, all patients were encouraged to ambulate using crutches with 25 to 35% of their body weight for 2 weeks and subsequently walk without crutches. K-wires and plates were removed at 1–2 years after surgery; all patients were followed up once annually using standard pelvic radiography and Harris scoring system at the outpatient department.

### Methods of assessment

**Fig. 2:**
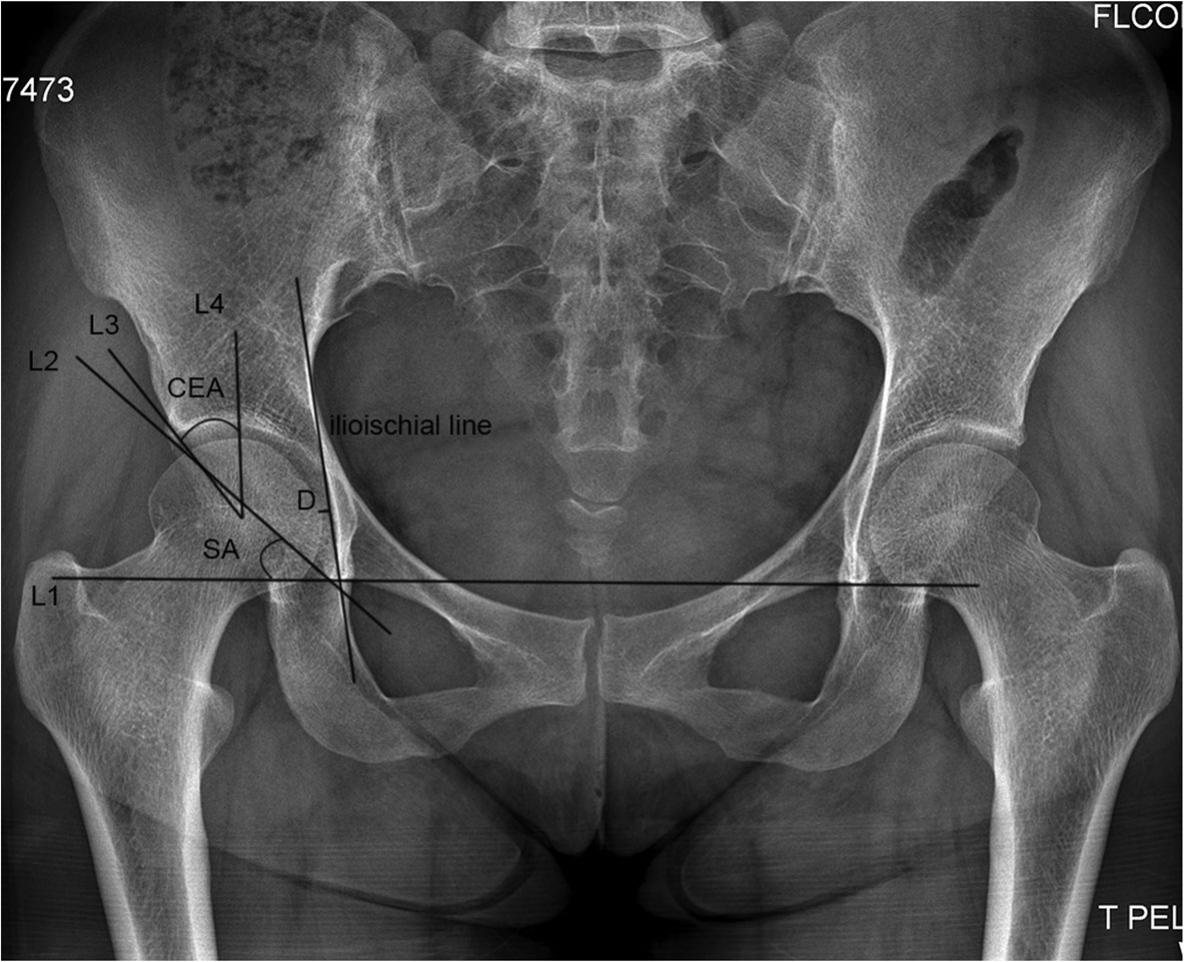
Diagram showing the radiographic parameters measured. L1 is the trans-teardrop line. L2 is the line between the teardrop and the acetabular edge. L3 is the line connecting the femoral head center and the acetabular edge. L4 is the vertical line at the femoral head center. The ilioischial line is the line connecting the lateral borders of the greater sciatic notch and obturator foramen. Sharp acetabular (SA) angle is the angle between L1 and L2, whereas center-edge angle (CEA) is the angle between L3 and L4. *D* is the distance between the medial femoral head and ilioischial line, which represents lateralization

All surgeries were performed by the senior author (CQZ). All standard anteroposterior pelvic radiographs before surgery and at the last follow-up were assessed by one author (JJW) who was blinded to the patient clinical status, and Harris hip scores were also analyzed (YY). Radiographic parameters included the Tönnis grade [13] used to evaluate osteoarthritic changes, Wiberg center-edge (CE) angle [14] used to determine the coverage of the anterior and middle/posterior areas of the femoral head, Sharp acetabular angle [15] used to determine the slopes of the anterior and middle/posterior portions of the acetabulum, and lateralization of the femoral head [16], which represents the distance from the medial edge of the femoral head to the ilioischial line (Fig. 2). According to preoperative Tönnis grade, patients were divided into group T0 (Tönnis grade 0), group T1 (Tönnis grade 1), and group T2 (Tönnis grade 2).

### Statistical analysis

Statistical analyses were performed using SPSS version 23 (IBM Corp., Armonk, NY, USA). Paired *t* test and chi-squared test were used to compare numeric variables and Tönnis grades, respectively, before the surgery and at the last follow-up in all patients. Multiple comparisons among the three groups were performed by least significant difference; *p* < 0.05 was considered statistically significant. Continuous variables were presented as mean ± standard deviation.

## Results

The mean age at surgery in all patients was 36.29 years (± 9.78) (range, 19–49) (Table 1), whereas that in groups T0, T1, and T2 was 31.08 (± 9.88) (range, 19–47), 37.30 (± 8.50) (range, 21–45), and 45.00 years (± 3.63) (range, 39–49), respectively (Table 2). Multiple comparisons among the three groups indicated that the mean age at surgery in group T2 was significantly higher than that in group T0 (*p* < 0.05) (Table 2). The right, left, and both sides were affected in 14, 10, and 2 patients, respectively. Their chief complaints were pain and limp. Patients were followed up for a mean of 8.93 years (± 1.94) (range, 6–13) (Table 1); in all patients, radiographic parameters significantly improved at the last follow-up compared with those preoperatively, with a 23.75° increase in CE angle, 16.18° decrease in Sharp acetabular angle, and 1.39 mm decrease in lateralization (*p* < 0.05) (Table 3). Multiple comparisons of radiographic outcomes among the three groups showed no significant difference at the last follow-up (Table 4). Further, there was no statistically significant difference in operative time and estimated operative blood loss in all groups (*p* > 0.05) (Table 1).

**Table 1:** General data of all patients

Item	Values	Range
Gender (male/female)	7/19	
Operative side (left/right)	12/16	
Age at surgery (years)	36.29 ± 9.78	19–49
Operation time (min)	102.50 ± 23.15	60–150
Estimated operative blood loss (ml)	805 ± 241.12	470–1350
Follow-up period (years)	8.93 ± 1.94	6–13

**Table 2:** General data of the three groups

Tönnis grade	Age (range, years)	Operative time (min)	Estimated operative blood loss (ml)
Group T0	31.08 ± 9.88 (19–47)	98.75 ± 17.21	801.67 ± 263.09
Group T1	37.30 ± 8.50 (21–45)	98.00 ± 24.29	842.00 ± 272.06
Group T2	45.00 ± 3.63^a^ (39–49)	117.50 ± 28.94	750.00 ± 148.46

^a^T2 vs. T0, *p* = 0.003. All values are expressed as mean ± standard deviation. Differences are considered significant at *p* < 0.05

**Table 3:** Preoperative and last follow-up results of radiographic parameters and Harris scores in 28 hips

Item	Before operation	Last follow-up	Improvement	*p* value
CE angle (°)	4.68 ± 3.99	28.43 ± 3.58	23.75	< 0.001
Sharp angle (°)	52.57 ± 5.73	36.39 ± 3.26	16.18	< 0.001
Lateralization (mm)	18.21 ± 4.89	16.82 ± 3.10	1.39	< 0.05
Harris score	73.71 ± 4.95	89.07 ± 4.97	15.36	< 0.001

All values are expressed as mean ± standard deviation. Differences are considered significant at *p* < 0.05

**Table 4:** Radiographic parameters of the three groups at different follow-up periods

Tönnis grade	CE angle (pre)	CE angle (last)	Sharp acetabular angle (pre)	Sharp acetabular angle (last)	Lateralization (pre)	Lateralization (last)
Group T0	4.33 ± 4.16	28.50 ± 4.17	50.58 ± 4.38	36.25 ± 3.31	16.83 ± 5.52	15.83 ± 3.71
Group T1	5.80 ± 4.66	27.90 ± 3.73	53.10 ± 5.36	35.70 ± 3.13	19.60 ± 3.84	17.30 ± 2.06
Group T2	3.50 ± 2.07	29.17 ± 2.23	55.67 ± 7.87	37.83 ± 3.49	18.67 ± 5.20	18.00 ± 3.10

CE, center edge; pre, preoperative; last, last follow-up. All values are expressed as mean ± standard deviation. Differences are considered significant at *p* < 0.05

The Harris scores in all patients improved from 73.71 (± 4.95) preoperatively to 89.07 (± 4.97) at the last follow-up, with variation in values being 15.36 (*p* < 0.05) (Table 3). Multiple comparisons showed significantly lower Harris scores in group T2 than in groups T0 and T1 at the last follow-up (*p* < 0.05) (Table 5).

**Table 5:** Harris scores preoperatively and at the last follow-up

Tönnis grade	Before operation	Last follow-up
Group T0	76.42 ± 4.89	91.75 ± 3.67
Group T1	72.60 ± 4.33	89.70 ± 3.86
Group T2	70.17 ± 3.31^a^	82.67 ± 3.08^b^

^a^Preoperatively—T2 vs. T0, *p* = 0.009; ^b^At the last follow-up—T2 vs. T0, *p* = 0.001; T2 vs. T1, *p* = 0.001. All values are expressed as mean ± standard deviation. Differences are considered significant at *p* < 0.05

No significant change in Tönnis grades preoperatively and at the last follow-up was observed (*p* > 0.05) (Table 6). Of the 28 hips, 12, 10, and 6 preoperatively had Tönnis grades 0, 1, and 2, respectively; more advanced osteoarthritis was not observed. In contrast, at the last follow-up, 13, 8, and 7 hips had Tönnis grades 0, 1, and 2, respectively, with no hip with Tönnis grade 3 or more advanced osteoarthritis having been observed. The Tönnis grade changed from grades 0 to 1 and from grades 1 to 2 in two hips.

**Table 6:** Tönnis grades for 28 hips preoperatively and at the last follow-up

Tönnis grade	Before operation, *n* (%)	Last follow-up, *n* (%)
Group T0	12 (42.86)	13 (46.43)
Group T1	10 (35.71)	8 (28.57)
Group T2	6 (21.43)	7 (25.00)

No nonunion at the iliac osteotomy site, lower extremity palsy, discrepancy in leg length longer than 2 cm, dislocation or posterior subluxation of the hip joint, deep wound infection, or deep vein thrombosis occurred. A superficial wound infection in the iliac region was treated in three patients by changing the dressings. No major complication was observed in the present study.

## Discussion

The relationship between the degree of radiographically detected dysplasia and development of osteoarthritis of the hip joint has been confirmed [17–19]. Acetabular dysplasia is generally characterized by a shallow acetabulum and malposition of the femoral head, leading to insufficient femoral head coverage on the anterior and superior lateral side. Thus, a small contact area between the femoral head and acetabulum increases the contact stress, resulting in sharply increased hip joint reaction forces beyond the cartilage stress threshold, which leads to cartilage deterioration and osteoarthritis of the hip joint [20].

The Steel triple pelvic osteotomy [2] was developed to increase the femoral head surface burdened by stress and medialize the femoral head by redistributing the increased contract stress from the ilium to the pubic and ischial rami, making the reorientation of the acetabulum possible. Several studies showed that the original Steel triple pelvic osteotomy [3, 6] or modified triple pelvic osteotomy [8, 10, 11] not only improved radiographic parameters but also relieved pain, led to satisfactory clinical results, and could retard osteoarthritis development in adults. However, owing to restrictions on strong ligamentous attachments in the original Steel triple pelvic osteotomy, it is difficult to achieve optimum coverage and medialization of the femoral head. The use of relatively nonrigid implants can result in nonunion and rehabilitation delay, and using three incisions makes the procedure complex. A posterior approach for ischial ramus osteotomy can pose a risk of sciatic nerve injury. The outcomes were unsatisfactory and suboptimal [21].

**Fig. 3:**
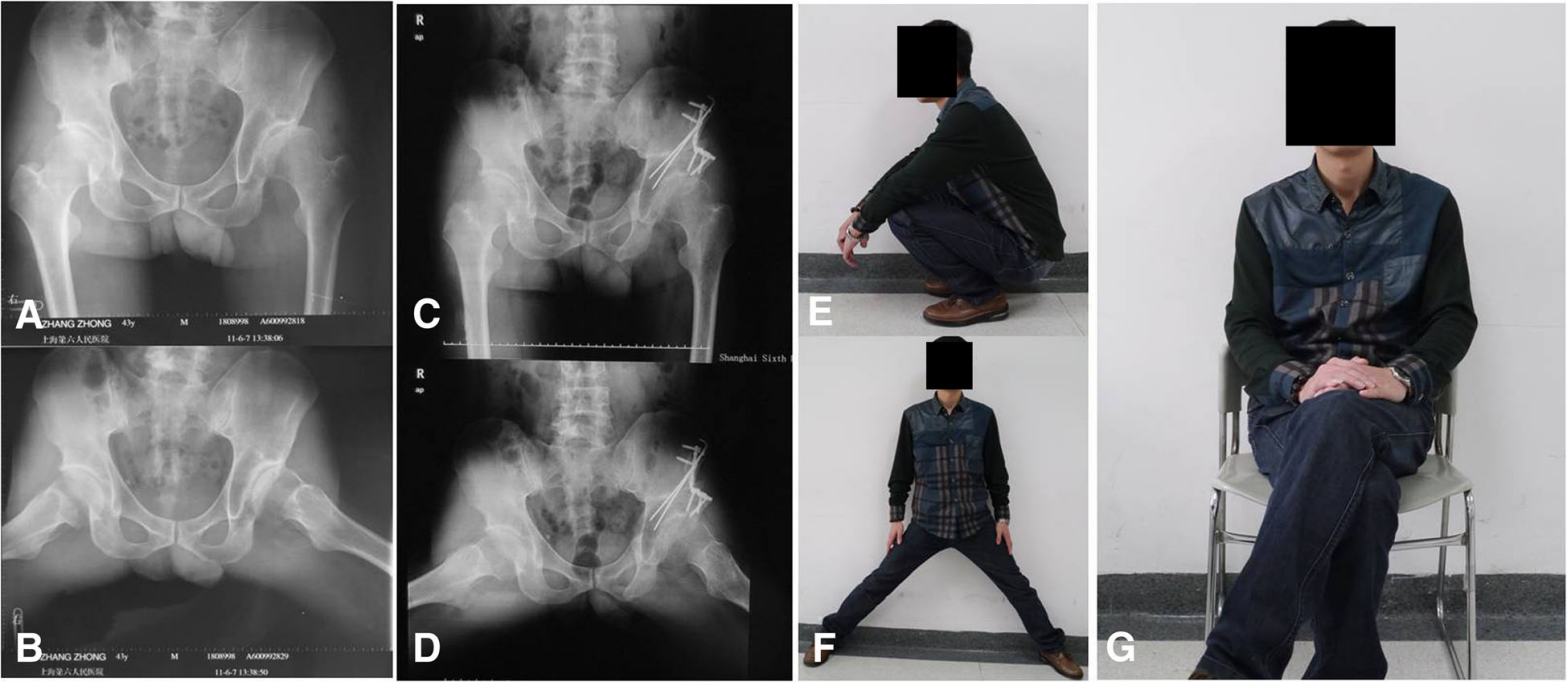
Radiographs of a 41-year-old male patient admitted for gradual pain in the left hip and limping for 8 months who was diagnosed with developmental dysplasia of the left hip (**a**) and Tönnis grade 1 osteoarthritis on radiography (**b**). Radiographs after modified triple pelvic osteotomy for the left hip was performed with the patient under general anesthesia (**c**) and at 6 months of follow-up (**d**). It was observed that the coverage rate of the femoral head significantly increased, the operative area healed well, pain and limping disappeared, and the range of hip motion improved. The range of hip motion of the same patient at 6 months after the surgery (**e**, **f**, **g**)

In our study, the mean CE angle improved by 23.75°, the mean Sharp acetabular angle decreased by 16.18°, and the lateralization increased by 1.39 mm at the last follow-up compared to those before surgery, suggesting that our new modified triple pelvic osteotomy not only results in better restoration of the normal anatomical characteristics of the acetabulum but also offers precise reorientation and stable fixation of the osteotomy site, achieving a satisfactory clinical outcome (Fig. 3).

Compared with the original Steel triple pelvic osteotomy, our simple modified anteromedial longitudinal approach at the proximal thigh could simultaneously expose both the pubic and ischial rami without involving the sciatic nerve; moreover, it was easier to achieve optimum coverage and medialization of the femoral head. The use of locking reconstruction plate and pins for fixation of the osteotomy site could lead to a rigid fixation of osteotomy fragments, maintain hip joint stability, promote early postoperative rehabilitation [8], and prevent nonunion of osteotomy fragments compared with the use of pins only, such as in the original Steel triple pelvic osteotomy. However, attention should be paid to the risk of injury to the femoral nerves and vessels. Moreover, our modified procedure simplified the surgical procedures without the need for intraoperative postural changes and could allow for the achievement of the goal of fast learning.

Triple pelvic osteotomy performed in younger patients with or without early-stage osteoarthritis is believed to result in almost normal orientation of the acetabulum and prevent or delay osteoarthritis development [9, 22]. To date, there are few studies in the literature that compared the clinical and radiographic results of patients with different osteoarthritis grades. In this study, our series of patients with Tönnis grade 0 or 1 had significantly improved clinical results compared with those with Tönnis grade 2 at different follow-up periods; it has been shown that patients with advanced osteoarthritis are more likely to have an unfavorable clinical outcome, which may imply that triple pelvic osteotomy is more suitable for patients with low-grade osteoarthritis than for patients with advanced osteoarthritis.

The limitations of the present study include its retrospective design, relatively small number of patients, and inherent difficulties in retrieving complete data. Moreover, radiographic measurements may vary from one observer to another, introducing a significant uncontrolled variable. In this study, no hip procedures were converted to total hip arthroplasty at a mean follow-up of 8.93 years, which was considered a relatively short follow-up period. The long-term clinical results of the procedure remain unknown, and further studies with longer follow-up periods are required to clarify this. However, we believe that our data provided evidence that our modified triple pelvic osteotomy can effectively delay the progression and exacerbation of symptomatic acetabular dysplasia.

## Conclusion

The performance of our modified triple pelvic osteotomy technique as a treatment for adult symptomatic acetabular dysplasia led to good clinical results, excellent radiographic outcomes, and lower complication rates. Younger patients with symptomatic acetabular dysplasia and low-grade osteoarthritis are more likely to have better clinical results. Long-term follow-up is necessary to determine the outcome of this modified procedure.

## Abbreviation

CE: Center-edge
